# Antimicrobial Drug–Resistant Bacteria Isolated from Syrian War–Injured Patients, August 2011–March 2013

**DOI:** 10.3201/eid2011.140835

**Published:** 2014-11

**Authors:** Carrie Lee Teicher, Jean-Baptiste Ronat, Rasheed M. Fakhri, Mohamed Basel, Amy S. Labar, Patrick Herard, Richard A. Murphy

**Affiliations:** Epicentre, New York, New York, USA (C.L. Teicher);; Médecins Sans Frontières/Doctors Without Borders, Paris, France (J.-B. Ronat, P. Herard);; Médecins Sans Frontières/Doctors Without Borders, Amman, Jordan (R.M. Fakhri, M. Basel);; Harvard School of Public Health, Boston, Massachusetts (A.S. Labar);; Médecins Sans Frontières/Doctors Without Borders, New York (R.A. Murphy);; Albert Einstein College of Medicine, Bronx, New York (R.A. Murphy)

**Keywords:** antimicrobial drug resistance, multidrug-resistant, MDR, bacteria, Syrian War, environmental contamination of wounds, Médecins Sans Frontières/Doctors Without Borders, Jordan, war-associated injuries

**To the Editor:** Soft-tissue injuries sustained during wars are subject to environmental contamination and, thus, to a high risk for infection. Efforts to describe the epidemiology of war-associated infections are complicated by difficult access to patients, limited availability of microbiology support, and widespread empirical antimicrobial drug use. Nevertheless, identifying the relevant pathogens is critical because war-associated injuries commonly become infected and antimicrobial drug–resistant bacteria are well-described in these injuries, including those in the Middle East (*1*–*3*).

The Médecins Sans Frontières (MSF) surgical project in Amman, Jordan, was initially developed for war-injured Iraqis needing surgical reconstruction or management of chronic osteomyelitis. Infection management is based on organism-directed antimicrobial agents and wide surgical resection of involved tissue. The proximity of this project to the Syrian conflict provided an opportunity to describe microbiologic features of infections caused by war-associated injuries in Syrians, who may be at increased risk for infection-associated complications because of exclusion from care in official health systems. We describe a cross-sectional series of 61 Syrian orthopedic patients who had suspected infections, as determined on the basis of surgical samples obtained intraoperatively.

Syrian patients admitted to the MSF clinic underwent initial surgical exploration of wounds; if infection was suspected, >3 intraoperative samples (bone, fibrous tissue, fluid) were obtained for culture and transported (at 4°–8°C) within 2 h to the laboratory at Ibn al-Haytham Hospital in Amman. Patients who were treated with antimicrobial drugs within 2 weeks before admission were excluded from analysis.

We retrospectively reviewed data for patients admitted during August 1, 2011–March 31, 2013. Data were collected from databases and individual charts in Amman and analyzed by using Stata 12 (http://www.stata.com/stata12/). This study was deemed exempt from additional ethical approval by the MSF review board because it involved routinely collected data.

We defined a multidrug-resistant (MDR) isolate as 1) extended-spectrum β-lactamase–expressing *Enterobacteriaceae*; 2) *Pseudomonas aeruginosa* and *Acinetobacter baumannii* isolates resistant to at least 1 agent in 3 antimicrobial categories typically used for treatment; or 3) methicillin-resistant *Staphylococcus aureus* (MRSA)*.* Pathogen identification was conducted by using conventional methods and the API system (bio-Mérieux, Durham, NC, USA). Antimicrobial drug susceptibility testing was conducted by using the MicroScan Walk-Away System (Dade Behring, West Sacramento, CA, USA).

During the study period, 870 patient consultations were conducted, of which 345 (40%) were for patients from Syria. At the initial operating room evaluation, infection was suspected in 61 (18%) Syrians. These patients had a median age of 26 years (interquartile range 22–34); 98% were male. The median time from injury to admission was 5 months (interquartile range 1.2–8.1), but for 27 (44%) patients, the time from injury to admission was >6 months. The 2 most common injuries were gunshot wounds (32 patients [52%]) and wounds from explosions (20 patients [33%]). The dominant injury was located in an upper extremity in 14 (23%) patients and a lower extremity in 47 (77%) patients.

For the 61 patients, a total of 67 bacterial isolates were identified from cultures of surgical specimens. Overall, 45 (74%) patients had at least 1 positive culture, and 6 (13%) patients had polymicrobial results. Gram-negative organisms represented 24 (56%) of 43 isolates; 10 (23%) were *P. aeruginosa*, 8 (19%) were *E. coli*, and 6 (14%) were *A. baumannii*. Gram-positive bacteria, including MRSA, represented 19 (44%) of 43 isolates (Table). Overall, 31 (69%) of 45 patients with confirmed infection were positive for MDR organisms. Within this group, MRSA represented 8 (42%) of 19 staphylococcal isolates.

**Table:** Antimicrobial drug resistance among frequently isolated bacterial isolates from Syrian patients with war-associated wound infections, August 2011–March 2013*

Antimicrobial drug	No. MDR resistant isolates/no. total (%)			
	*Staphylococcus aureus*, N = 19	*Pseudomonas aeruginosa*, N = 10	*Escherichia coli*, N = 8	*Acinetobacter baumannii*, N = 6
Amikacin		1/ 11 (9)	1/7 (14)	6/6 (100)
Ampicillin			5/5 (100)	
Amoxicillin/clavulanic acid			6/6 (100)	
Cefotaxime			6/8 (75)	
Ceftriaxone			5/8 (62)	
Ceftazidime		3/9 (33)	5/8 (62)	4/4 (100)
Cefepime			5/8 (62)	5/5 (100)
Cefixime			5/8 (62)	5/5 (100)
Ciprofloxacin	7/17 (41)	5/8 (62)	2/7 (28)	5/5 (100)
Colistin		NA	NA	0/5
Trimethoprim/sulfamethoxazole	3/14 (21)		3/5 (60)	
Gentamicin	10/18 (55)	4/9 (44)	4/8 (50)	6/6 (100)
Piperacillin/tazobactam		2/9 (22)	3/7 (42)	NA
Imipenem		0/9	1/7 (14)	4/5 (80)
Penicillin	9/10 (90)			
Oxacillin	7/17 (41)			
Clindamycin	9/17 (52)			
Rifampin	6/15 (40)			
Fusidic acid	10/15 (66)			

*Blank cells indicate that testing was not done.

Patients who had experienced delayed definitive management were frequently positive for MDR organisms, especially gram-negative pathogens and MRSA. For a humanitarian surgical project, infection with MDR organisms leads to formidable diagnostic, treatment, and control challenges. For example, treatment of MDR infections requires ongoing access to high-quality clinical microbiology support; late-generation antimicrobial drugs, which are typically given parenterally for up to 6 weeks; trained personnel; and sufficient hospital space to isolate patients with resistant strains. Our findings support the previously reported linkage between war-associated injuries and infection with antimicrobial drug–resistant organisms (*1*–*4*) and the implications for patient management.

The source of antimicrobial drug–resistant organisms in war-associated injuries remains uncertain; possibilities include nosocomial transmission (*5*), particularly through prior contact with severely compromised health systems (*6*). Another possibility is fecal colonization with extended-spectrum β-lactamase–producing gram-negative bacteria. (*7*,*8*). Another likely contributor in Syria is the wide availability of antimicrobial drugs without a prescription (*9*).

This study has limitations. Although measures were taken to ensure that positive cultures represented clinical infection rather than colonization, we cannot exclude colonization as a possible source of some recovered organisms. In neglected war-associated injuries, multiple pathogens are potentially present, but every strain is not necessarily clinically relevant (*10*). Furthermore, complete patient histories are difficult to obtain in crisis settings, limiting our ability to describe all prior interventions. Study strengths included partnership with a high-quality culture laboratory, which is uncommon in programs treating war injuries; systemic sampling of patients with suspected infection; and use of intraoperative samples for culture. Further research needed in this neglected area includes prospective studies to determine the effect of MDR isolates on patient outcomes and randomized clinical trials of antimicrobial drug strategies to inform treatment protocols.

## References

[1] Franka EA, Shembesh MK, Zaied AA, El-Turki E, Zorgani A, Elahmer OR, Multidrug resistant bacteria in wounds of combatants of the Libyan uprising. J Infect. 2012;65:279–81 . Epub 2012 Apr 7. 10.1016/j.jinf.2012.04.002 22490615

[2] Sutter DE, Bradshaw LU, Simkins LH, Summers AM, Atha M, Elwood RL, High incidence of multidrug-resistant gram-negative bacteria recovered from Afghan patients at a deployed US military hospital. Infect Control Hosp Epidemiol. 2011;32:854–60. 10.1086/661284 21828965

[3] Murray CK, Griffith ME, Mende K, Guymon CH, Ellis MW, Beckius M, Methicillin-resistant *Staphylococcus aureus* in wound cultures recovered from a combat support hospital in Iraq. J Trauma. 2010;69(Suppl 1):S102–8. 10.1097/TA.0b013e3181e44b57 20622603

[4] Rafei R, Dabboussi F, Hamze M, Eveillard M, Lemarié C, Mallat H, First report of bla_NDM-1_–producing *Acinetobacter baumannii* isolated in Lebanon from civilians wounded during the Syrian war. Int J Infect Dis. 2014;21:21–3. Epub 2014 Feb 19. 10.1016/j.ijid.2014.01.004 24560830

[5] Peretz A, Labay K, Zonis Z, Glikman D. Disengagement does not apply to bacteria: a high carriage rate of antibiotic-resistant pathogens among Syrian civilians treated in Israeli hospitals. Clin Infect Dis. Epub 2014 May 20. PubMed 10.1093/cid/ciu374 24846634

[6] Murphy RA, Ronat JB, Fakhri RM, Herard P, Blackwell N, Abgrall S, Multidrug-resistant chronic osteomyelitis complicating war injury in Iraqi civilians. J Trauma. 2011;71:252–4. 10.1097/TA.0b013e31821b8622 21818032

[7] Kader AA, Kumar A, Kamath KA. Fecal carriage of extended-spectrum β-lactamase–producing *Escherichia coli* and *Klebsiella pneumoniae* in patients and asymptomatic healthy individuals. Infect Control Hosp Epidemiol. 2007;28:1114–6. Epub 2007 Jun 28. 10.1086/519865 17932839

[8] Vento TJ, Cole DW, Mende K, Calvano TP, Rini EA, Tully CC, Multidrug-resistant gram-negative bacteria colonization of healthy US military personnel in the US and Afghanistan. BMC Infect Dis. 2013;13:68. 10.1186/1471-2334-13-68 23384348 PMC3610270

[9] Al-Faham Z, Habboub G, Takriti F. The sale of antibiotics without prescription in pharmacies in Damascus, Syria. J Infect Dev Ctries. 2011;5:396–9. 21628818 10.3855/jidc.1248

[10] Johnson EN, Burns TC, Hayda RA, Hospenthal DR, Murray CK. Infectious complications of open type III tibial fractures among combat casualties. Clin Infect Dis. 2007;45:409–15 . Epub 2007 Jul 5. 10.1086/520029 17638186

